# Targeted delivery and controlled release of deferasirox for melanoma therapy

**DOI:** 10.1016/j.isci.2026.115303

**Published:** 2026-03-10

**Authors:** Xiaochen Su, Weitao Zhao, Lulu Wang, Panpan Song, Xingbo Wang, Xuefei Jin, Haiyuan Zhang

**Affiliations:** 1 Second Inpatient Area of Urology Department, China–Japan Union Hospital of Jilin University, Changchun, China; 2 School of Pharmaceutical Sciences, Jilin University, Changchun, China; 3 Laboratory of Chemical Biology, Changchun Institute of Applied Chemistry, Chinese Academy of Sciences, Changchun, China; 4 School of Biomedical Engineering and the First Affiliated Hospital, Guangzhou Medical University, Guangzhou, China

**Keywords:** cancer, therapy

## Abstract

Melanoma, the most lethal form of skin cancer, remains a significant therapeutic challenge despite advances in immunotherapy. Although PD-1/PD-L1 blockade improves clinical outcomes, its effectiveness is frequently limited by suboptimal response rates and treatment resistance. Here, we developed a novel strategy targeting iron-dependent PD-L1 regulation. Since elevated iron activates phosphoinositide-3-kinase (PI3K)/AKT signaling and upregulates PD-L1, we employed the iron chelator deferasirox (DFX) to disrupt this pathway. To overcome DFX’s poor solubility and bioavailability, we engineered tumor-targeting, glutathione-responsive albumin nanoparticles modified with cRGD peptides (_RGD_AB@DFX NPs). These NPs selectively accumulated in B16F1 melanoma tumors and released DFX in response to intracellular glutathione, effectively downregulating PD-L1. These findings suggest that iron modulation represents a promising approach to enhance immunotherapy efficacy, with _RGD_AB@DFX NPs serving as an optimized delivery platform for clinical application.

## Highlights

• _RGD_AB@DFX NPs were developed to responsively release DFX in B16F1 melanoma cells
• Released DFX chelated iron and reduced PD-L1 expression via the PI3K/AKT pathway
• Tumor growth in B16F1 mouse model was significantly inhibited by these NPs
• The therapeutic effect was related to iron-chelated enhanced antitumor immunity

## Graphical abstract

**Figure:**
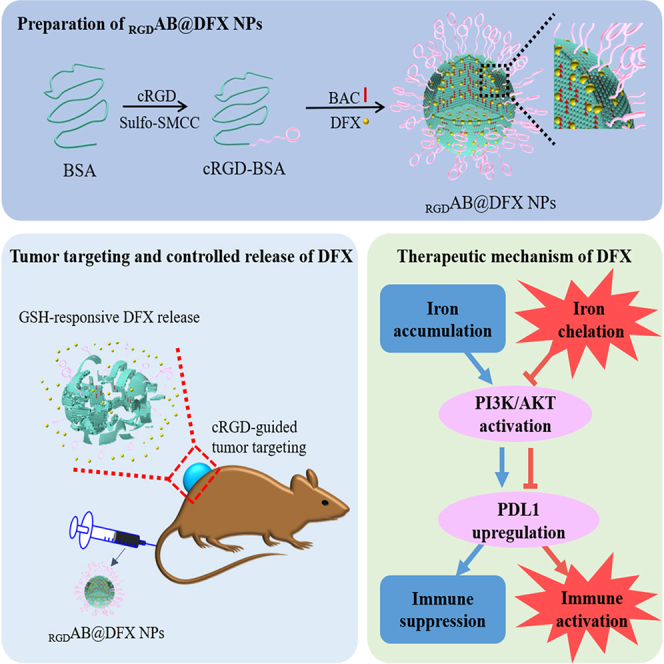

## Introduction

Melanoma is the most fatal form of skin cancer, characterized by a high recurrence rate after clinical treatment.^1^^,^^2^ Historically, few effective treatments have been available for advanced melanoma.^3^ The introduction of immunotherapy has dramatically improved survival of patients with melanoma.^4^^,^^5^ Among these, immune checkpoint blockade (ICB) therapy has been widely applied in clinical cancer treatment, showing great potential.^6^ In particular, therapeutic antibodies targeting programmed cell-death protein 1 (PD-1)/programmed cell-death 1 ligand 1 (PD-L1) axis, such as nivolumab, pembrolizumab and BMS-936559, have shown remarkable efficacy against melanoma.^7^ Nevertheless, the PD-1/PD-L1 blockade therapy remains limited by low response rates in certain cancers, lack of reliable biomarkers, immune-related toxicities and both innate and acquired drug resistance.^8^ To date, the clinical response to PD-1/PD-L1 blockade is about 40%.^9^ Therefore, the development of new strategies to enhance the immunotherapy efficacy remains a critical area of investigation.

Downregulation of PD-L1 expression in tumor microenvironment (TME) is emerging as new approach to improve immunotherapy efficacy.^10^^,^^11^^,^^12^ The phosphoinositide-3-kinase (PI3K)/protein kinase B (AKT) signaling pathway plays an important role in regulating signal transduction and biological processes such as proliferation, apoptosis, metabolism and angiogenesis.^13^^,^^14^ Inhibition of PI3K-AKT pathway has been found to downregulate PD-L1 expression.^11^^,^^15^^,^^16^ Iron (Fe), an important element for numerous physiological processes, participates in cellular energy mechanism and the synthesis of genetic materials. It is indispensable throughout the whole stage of tumor development, survival, proliferation, and metastasis.^17^^,^^18^ Fe element can also affect the expression of multiple gene involved in cellular activity. In tumor cells, due to changes in metabolism, the Fe content increases compared with that of normal cells. Recent studies have demonstrated that high Fe content in cells can activate the PI3K/AKT pathway, accompanied by an increased risk of cancer.^19^ Moreover, the activation of PI3K/AKT pathway has been associated with upregulated PD-L1 expression.^20^ This finding suggests that the reduction of the Fe content in tumor cells probably can decrease the expression of PD-L1 in tumor cells. Fe chelators, which have a strong affinity for Fe and can selectively bind it, are widely used to treat Fe overload disorders. Among U.S. Food and Drug Administration (FDA)-approved agents for anemia-associated chronic Fe overload, deferasirox (DFX) exhibits superior metal selectivity, circumventing deferiprone (DFP)’s weak Fe-binding affinity and deferoxamine (DFO)-mediated off-target chelation of other trivalent metals (e.g., aluminum ion (Al^3+^) and gallium ion (Ga^3+^)); coupled with its markedly prolonged half-life and the absence of auditory or ocular toxicity, DFX demonstrates a favorable clinical safety profile.^21^^,^^22^ Therefore, DFX potentially can be used to reduce the Fe content in tumor cells and down-regulate the PD-L1 expression, enhancing cancer immunotherapy. However, the poor water solubility, low bioavailability, and limited tumor-targeting ability of DFX restrict its medical applications.

Drug delivery strategy can significantly improve the stability, bioavailability, tumor-targeting ability, and controlled release of drug. In the present study, tumor-targeting, glutathione (GSH)-cleavable and DFX-loaded albumin nanoparticles (_RGD_AB@DFX NPs) were prepared to deliver DFX to tumor tissues and improve DFX-mediated tumor therapy. N, N′-bis (acryloyl) cystamine (BAC) as disulfide-bond-containing cross-linker was embedded in DFX-loaded A NPs (A@DFX NPs) to form AB@DFX NPs, followed by surface modification with cyclic arginine-glycine-aspartic acid (cRGD) peptides to yield _RGD_AB@DFX NPs. Through intravenous injection into B16F1 melanoma-bearing mice, _RGD_AB@DFX NPs could specifically accumulate at tumor tissues, be internalized by tumor cells, and respond to high intracellular GSH levels to cleave disulfide bond and release DFX, thereby reducing PD-L1 expression and improving antitumor therapy.

## Results

### Physicochemical property of A, A@DFX, AB@DFX and _RGD_AB@DFX NPs

**Figure 1:**
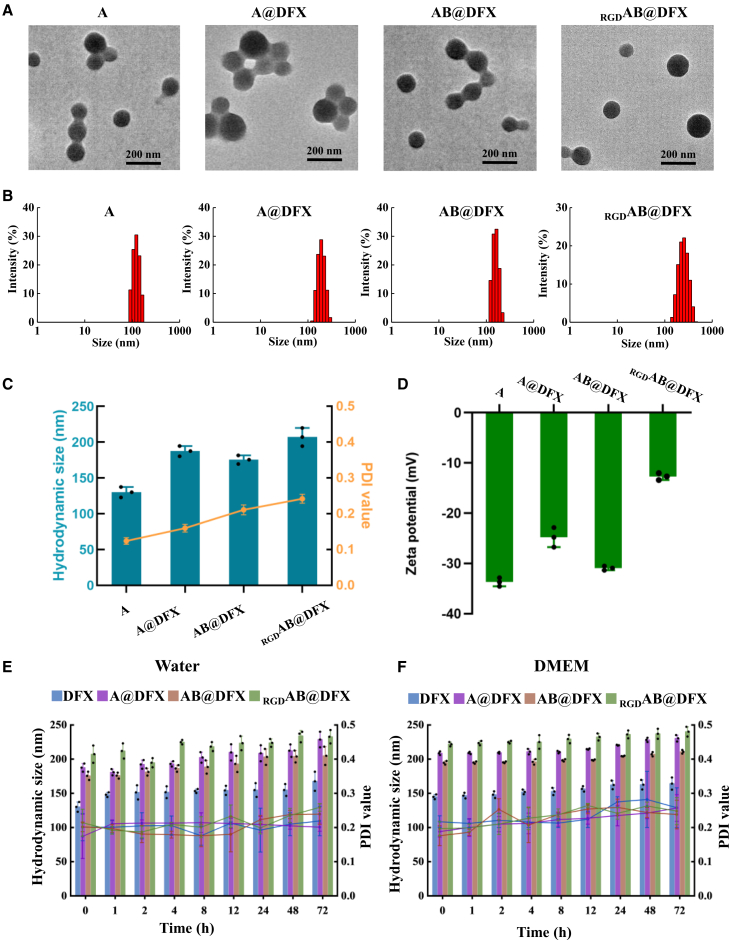
Characterization of A, A@DFX, AB@DFX, and _RGD_AB@DFX NPs (A) TEM image. Scale bars, 200 nm. (B) Primary size. (C) Hydrodynamic size and PDI. Data are expressed as means ± SD (*n* = 3 independent biological replicates). (D) Zeta potential. Data are expressed as means ± SD (*n* = 3 independent biological replicates). (E) Hydrodynamic sizes and PDI values in water over 72 h incubation. Data are expressed as means ± SD (*n* = 3 independent biological replicates). (F) Hydrodynamic sizes and PDI values in DMEM containing 10% serum over 72 h incubation. Data are expressed as means ± SD (*n* = 3 independent biological replicates).

A, A@DFX, AB@DFX and _RGD_AB@DFX NPs were prepared using a desolvation method, and their size and morphology were characterized by transmission electron microscope (TEM). Figure 1 shows all the NPs exhibited spherical morphology with smooth surfaces (Figure 1A), and their primary sizes were 97.6 ± 10.8, 124.8 ± 22.2, 100.2 ± 17.5 and 116.8 ± 22.0 nm for A, A@DFX, AB@DFX and _RGD_AB@DFX NPs, respectively (Figure 1B). Figure 1C shows the hydrodynamic sizes of A, A@DFX, AB@DFX and _RGD_AB@DFX NPs in deionized water were 130.1 ± 7.2, 187.5 ± 7.2, 175.5 ± 6.0 and 207.2 ± 12.6 nm, respectively, with corresponding polydispersity index (PDI) of 0.12, 0.16, 0.21 and 0.24, respectively. These results indicate that the DFX loading results in the increased particle sizes in A@DFX and AB@DFX NPs compared with A NPs, while cRGD modification can further enlarge the particle size of _RGD_AB@DFX NPs compared with AB@DFX NPs. The elevation in hydrodynamic size of _RGD_AB@DFX NPs compared with AB@DFX NPs is attributed to cRGD modification on BSA. On one hand, cRGD peptide modification increases the molecular length of BSA, resulting in increased physical sizes of _RGD_AB@DFX NPs, and on the other hand, cRGD on the particle surface introduces an additional hydration layer, resulting in increased hydrodynamic sizes of _RGD_AB@DFX NPs. Figure 1D shows the zeta potentials of A, A@DFX, AB@DFX and _RGD_AB@DFX NPs were −33.7 ± 0.9, −24.8 ± 1.9, −30.9 ± 0.4 and −10.7 ± 0.9 mV, respectively. The DFX loading slightly increased the surface charges of A@DFX, AB@DFX NPs compared with A NPs, and cRGD modification further markedly increased the surface charge of _RGD_AB@DFX NPs compared with AB@DFX NPs. The positive shift in zeta potential of _RGD_AB@DFX NPs compared with AB@DFX NPs is attributed to the positively charged guanidine group of arginine residues of cRGD on the particle surface. The stability of various NPs was evaluated through monitoring their hydrodynamic size variation over 72 h of incubation in water and Dulbecco’s Modified Eagle Medium (DMEM) containing 10% serum. Figures 1E and 1F indicate that these NPs maintained stable hydrodynamic sizes in both media, with the slightly larger sizes observed in DMEM, suggesting their robust stability. Moreover, the DFX loading capacities of A@DFX NPs, AB@DFX NPs and _RGD_AB@DFX NPs were 17.4% ± 2.0%, 16.7% ± 1.2% and 16.3% ± 1.7%, respectively, as determined by HPLC.

### GSH-responsive drug release profiles of _RGD_AB@DFX NPs

**Figure 2:**
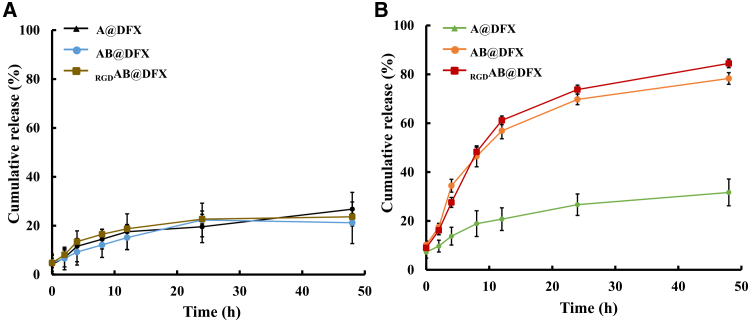
GSH-responsive DFX release profiles of A@DFX, AB@DFX and _RGD_AB@DFX NPs (A) GSH-responsive DFX release profiles under 20 μmol L^−1^ GSH condition. Data are expressed as means ± SD (*n* = 3 independent biological replicates). (B) GSH-responsive DFX release profiles under 10 mmol L^−1^ GSH condition. Data are expressed as means ± SD (*n* = 3 independent biological replicates).

GSH-responsive drug release behavior of _RGD_AB@DFX NPs was evaluated in phosphate buffered saline (PBS) buffer under high (10.0 mmol L^−1^) or low (20 μmol L^−1^) GSH condition. Figure 2A shows that within 48 h, the cumulative release rates of A@DFX NPs, AB@DFX NPs and _RGD_AB@DFX NPs under 20 μmol L^−1^ GSH condition were 26.8 ± 6.9%, 21.2 ± 1.3% and 23.6 ± 8.5%, respectively, indicating minimal DFX release under extracellular condition. However, under 10.0 mmol L^−1^ GSH that simulates intracellular condition, the cumulative release rates of AB@DFX NPs and _RGD_AB@DFX NPs markedly increased to 78.3 ± 2.4% and 84.4 ± 1.8%, respectively, while A@DFX NPs maintained a low release rate of 31.7 ± 5.5% (Figure 2B). These results confirm that both AB@DFX NPs and _RGD_AB@DFX NPs exhibit GSH-responsive DFX release, attributable to the cleavage of disulfide bonds in BAC crosslinkers.

### Enhanced cellular uptake and PD-L1 suppression by _RGD_AB@DFX NPs in B16F1 cells

**Figure 3:**
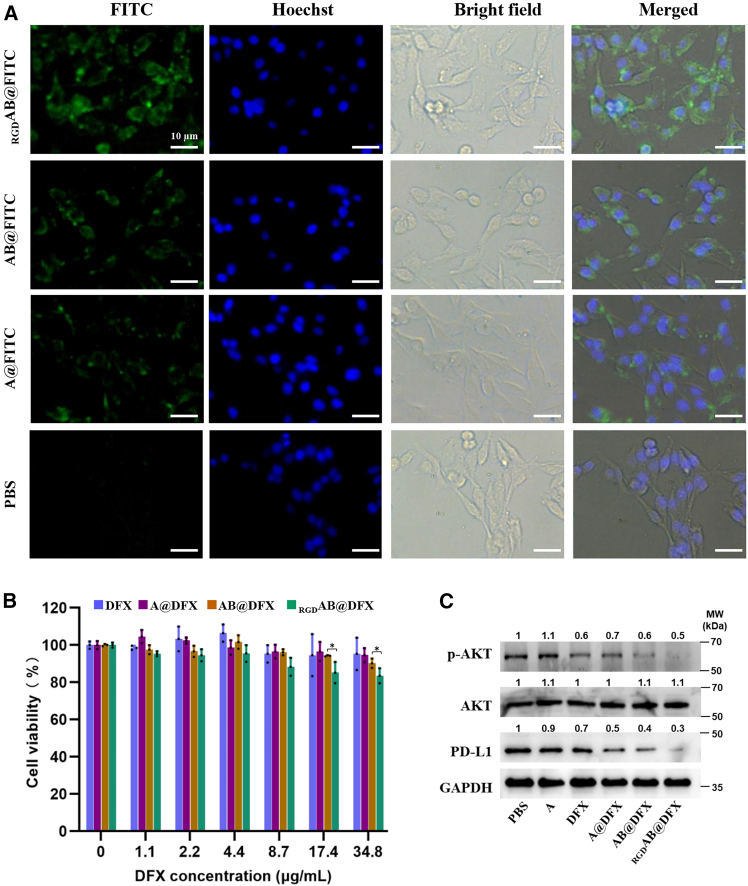
B16F1 cellular behaviors of A@DFX, AB@DFX and _RGD_AB@DFX NPs (A) Fluorescence microscopy images of cells treated with various FITC-labeled NPs for 6 h. Scale bars, 10 μm. (*n* = 3 independent biological replicates). (B) Cell viability assessment after 24 h of incubation with cells by MTT assay. Data are expressed as means ± SD (*n* = 3 independent biological replicates). Statistical analysis: one-way ANOVA followed by Tukey’s post hoc test. ∗*p* < 0.05. (C) Western blot analysis of *p*-AKT and PD-L1 expression of cells treated with various NPs for 6 h (*n* = 3 independent biological replicates).

Cellular uptake of A NPs, A@DFX NPs, AB@DFX NPs and _RGD_AB@DFX NPs in B16F1 cell was evaluated by fluorescence microscopy using FITC-labeled NPs. Figure 3A shows that cells treated with various FITC-labeled NPs exhibited higher green fluorescence than those with PBS, where FITC-labeled _RGD_AB@DFX NPs (_RGD_AB@DFX-FITC NPs) presented the highest fluorescence intensity due to their cRGD modification. Cytotoxicities of A NPs, A@DFX NPs, AB@DFX NPs and _RGD_AB@DFX NPs against B16F1 cells were assessed using the MTT assay. After 24 h of incubation, it was found that the cell viability was minimally affected, showing only a slight reduction of 15∼17% at the higher concentrations of _RGD_AB@DFX NPs (Figure 3B), suggesting the low cytotoxicity of _RGD_AB@DFX NPs. To further investigate the biological effects, *p*-AKT and PD-L1 expressions of cells were further assessed by western blot analysis. Figure 3C indicates that *p*-AKT and PD-L1 expressions of cells treated with PBS showed high levels, and treatment with A NPs almost did not alter the *p*-AKT and PD-L1 expression levels. However, treatments with DFX, A@DFX NPs, AB@DFX NPs and _RGD_AB@DFX NPs could reduce the *p*-AKT and PD-L1 expression levels, with _RGD_AB@DFX NPs exhibiting the most pronounced reduction. Taken together, these results indicate that _RGD_AB@DFX NPs could be efficiently internalized into B16F1 cells, where they respond to intracellular high GSH level to release DFX. These released DFX chelates Fe ions and downregulate *p*-AKT expression, leading to reduced PD-L1 expression. The weak cytotoxicity of _RGD_AB@DFX NPs is potentially ascribed to PD-L1 reduction.

### Enhanced tumor accumulation and prolonged circulation of _RGD_AB@DFX NPs for improved antitumor therapy

**Figure 4:**
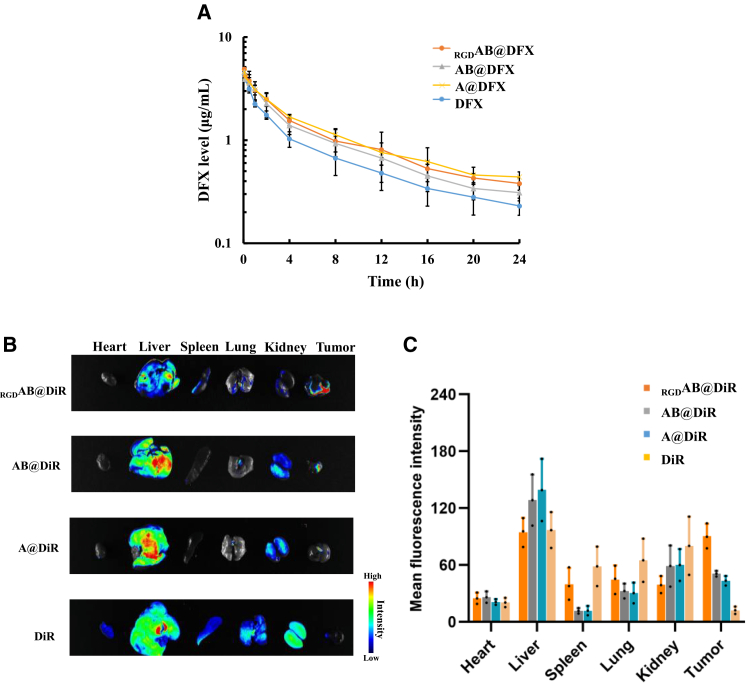
Pharmacokinetics and biodistribution of various NPs in B16F1 tumor-bearing mice (A) Plasma concentration-time curve of DFX of mice intravenously injected with DFX, A@DFX NPs, AB@DFX NPs and _RGD_AB@DFX NPs during 24 h. Data are expressed as means ± SD (*n* = 3 mice). (B) *Ex vivo* fluorescence images of tumor and major organs (heart, liver, spleen, lung, and kidney) of mice intravenously injected with DiR-labeled NPs (_RGD_AB@DiR NPs, AB@DiR NPs and A@DiR NPs) and DiR; the fluorescence images were collected at 24 h post-injection (*n* = 3 mice). (C) Quantitative fluorescence intensity analysis in (B). Data are expressed as means ± SD (*n* = 3 mice).

Encouraged by the significant PD-L1 reduction induced by _RGD_AB@DFX NPs *in vitro*, *in vivo* therapeutic efficacy of _RGD_AB@DFX NPs was further investigated in B16F1 tumor-bearing mice. Pharmacokinetics of DFX, A@DFX NPs, AB@DFX NPs and _RGD_AB@DFX NPs was first evaluated by HPLC. Compared with free DFX, all DFX-loaded NPs (A@DFX NPs, AB@DFX NPs and _RGD_AB@DFX NPs) maintained higher concentration over 24 h (Figure 4A). The averaged half-life (t_1/2_) of DFX in A@DFX NPs, AB@DFX NPs and _RGD_AB@DFX NPs was 11.209, 11.063 and 11.204 h, respectively, which were significantly longer than that (8.383 h) of free DFX. These results indicated that DFX encapsulation into NPs reduces the clearance rate of DFX and prolongs its circulation time. Then, *in vivo* biodistribution was evaluated using various DiR-labeled NPs. B16F1 tumor-bearing mice with tumor volumes of 100 mm^3^ were intravenously injected with various DiR-labeled NPs. At 24 h post-injection, the tumor tissues and major organs (heart, liver, spleen, lung, kidney) were harvested for fluorescence imaging. Figure 4B shows that all the DiR-labeled NPs had predominant accumulation in liver, while free DiR mainly accumulated at liver and kidney. Notably, DiR-labeled NPs showed visible accumulation in tumor tissues, with DiR-labeled _RGD_AB@DFX NPs exhibiting the strongest fluorescence signal. Further quantitative fluorescence intensity analysis supported this result (Figure 4C).

### _RGD_AB@DFX NPs suppress tumor growth via PD-L1 downregulation in B16F1 tumor-bearing mice

**Figure 5:**
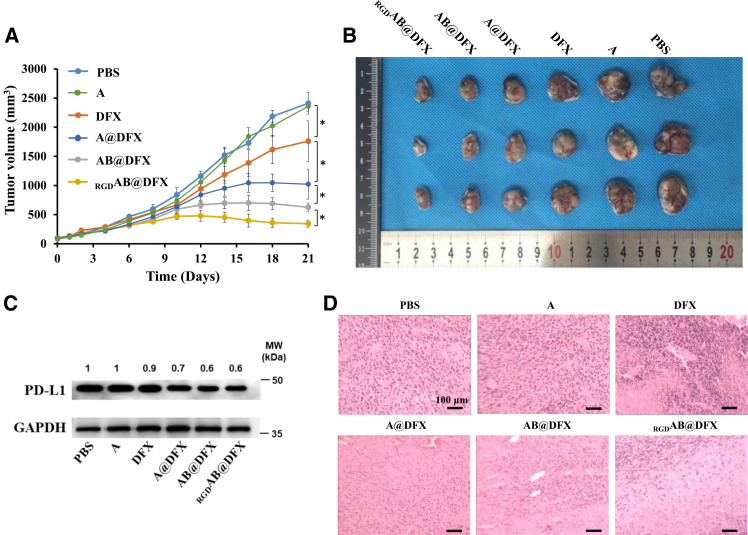
*In vivo* therapeutic effect of _RGD_AB@DFX NPs in B16F1 tumor-bearing mice (A) Tumor growth curves during 21 days of treatment with PBS, A NPs, DFX, A@DFX NPs, AB@DFX NPs and _RGD_AB@DFX NPs. Data are expressed as means ± SD (*n* = 3 mice). Statistical analysis: one-way ANOVA followed by Tukey’s post hoc test. ∗*p* < 0.05. (B) Photographs of tumors at the end of 21 days of various treatments (*n* = 3 mice). (C) PD-L1 expression of tumor tissues at the end of 21 days of various treatments (*n* = 3 independent biological replicates). (D) H&E staining of tumor tissue sections at the end of 21 days of various treatments. Scale bars, 100 μm.

Antitumor therapeutic effects of _RGD_AB@DFX NPs were further evaluated through monitoring tumor growth, analyzing PD-L1 expression and observing pathological changes. When the tumor volumes reached approximately 100 mm^3^, B16F1 tumor-bearing mice were randomly divided into six groups as follows: (1) PBS, (2) A NPs, (3) DFX, (4) A@DFX NPs, (5) AB@DFX NPs, and (6) _RGD_AB@DFX NPs. Mice were intravenously injected with the respective formulations every other day for a total of three doses. Tumor volumes of mice were measured every other day. As shown in Figure 5A, tumor growth in mice treated with A NPs was comparable to that of the PBS control group. Treatment with free DFX slightly inhibited tumor growth compared with PBS. However, A@DFX NPs, AB@DFX NPs, and _RGD_AB@DFX NPs all demonstrated significantly enhanced tumor growth inhibition compared with free DFX, likely due to the enhanced permeability and retention (EPR) effect of NPs. Among them, AB@DFX NPs exhibited stronger tumor growth inhibition than A@DFX NPs, probably because of their more efficient DFX release ability within tumor cells. _RGD_AB@DFX NPs further achieved the most pronounced tumor suppression, which can be attributed to their superior tumor-targeting capability mediated by cRGD modification. Representative tumor images collected at the end of 21 days of treatments are shown in Figure 5B, and hematoxylin and eosin (H&E) staining results are presented in Figure 5C. Consistent with the tumor growth inhibition trend, _RGD_AB@DFX NPs induced the most extensive necrosis, followed by AB@DFX NPs, A@DFX NPs and DFX. The superior therapeutic performance of _RGD_AB@DFX NPs is likely ascribed to downregulated PD-L1 expression, which probably was triggered by DFX-mediated Fe chelation. At the end of treatments, PD-L1 expression of tumor tissues was further evaluated by western blot analysis. Figure 5D shows that treatment with _RGD_AB@DFX NPs resulted the lowest PD-L1 expression, followed by AB@DFX NPs, A@DFX NPs and DFX. These results are consistent with the observed tumor inhibition and histological outcomes. Collectively, these results demonstrate that _RGD_AB@DFX NPs can effectively suppress tumor growth through a DFX-mediated PD-L1 reduction mechanism.

### _RGD_AB@DFX NPs exhibit excellent biocompatibility and safety *in vivo*

**Figure 6:**
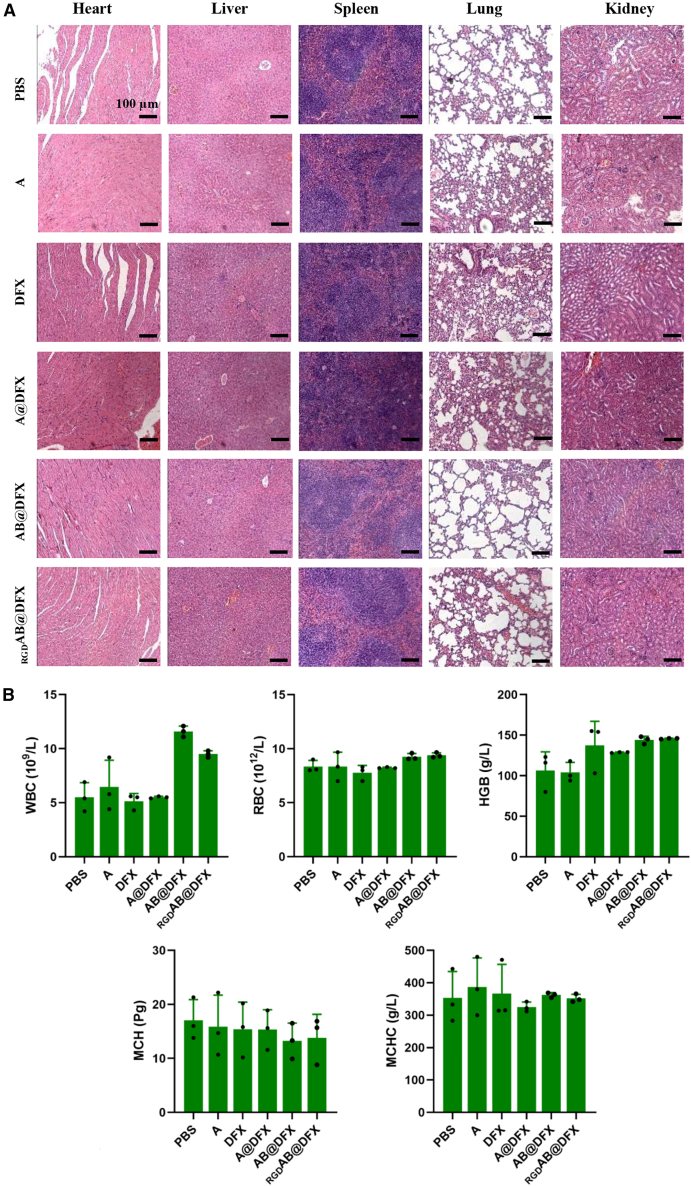
Biocompatibility of _RGD_AB@DFX NPs in B16F1 tumor-bearing mice (A) H&E staining of major organs (heart, liver, spleen, lung and kidney) at the end of 21 days of treatments with PBS, A NPs, DFX, A@DFX NPs, AB@DFX NPs and _RGD_AB@DFX NPs. Scale bars, 100 μm. (B) Blood routine indices of mice at the end of 21 days of various treatments. Data are expressed as means ± SD (*n* = 3 mice).

Biocompatibility was evaluated by monitoring body weight during the treatments, observing the pathological changes, and analyzing hematological and biochemical parameters at the end of treatment. Supporting information Figure S2 reveals that treatment with DFX and DFX-loaded NPs caused negligible changes in body weight of mice. H&E staining of major organs (heart, liver, spleen, lung and kidney) revealed no noticeable pathological abnormalities (Figure 6A). Moreover, hematological parameters, including white blood cells (WBCs), red blood cells (RBCs), hemoglobin (HGB), mean corpuscular hemoglobin (MCH) and mean corpuscular hemoglobin concentration (MCHC), remained within normal ranges across all the treatment groups (Figure 6B). Supporting information Figure S3 further indicates that levels of the liver function markers (alanine transaminase (ALT) and aspartate aminotransferase (AST)) and kidney function (blood urea nitrogen (BUN) and creatinine (CRE) were all within the physiological range. All above results demonstrate that _RGD_AB@DFX NPs has excellent biocompatibility and potent therapeutic efficacy *in vivo*.

### _RGD_AB@DFX NPs activate antitumor immunity by modulating the PD-1/PD-L1 axis

**Figure 7:**
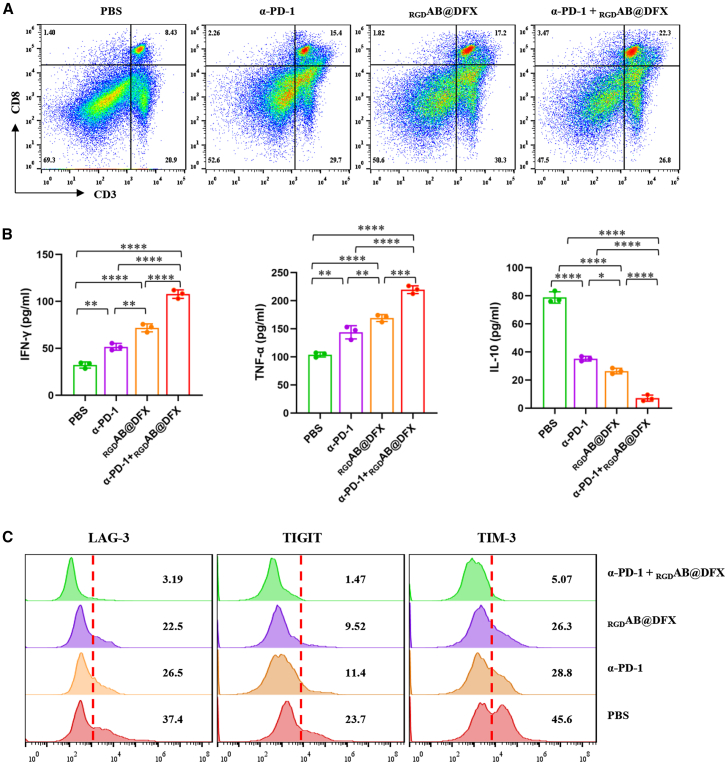
Mechanism validation of _RGD_AB@DFX NPs-mediated antitumor immunity (A) Flow cytometry data of CD8^+^ T cells in tumor tissues from different treatment groups. Data are expressed as means ± SD (*n* = 3 mice). (B) ELISA results of immune cytokines in tumor tissues from different treatment groups. Data are expressed as means ± SD (*n* = 3 mice). Statistical analysis: one-way ANOVA followed by Tukey’s post hoc test. ∗*p* < 0.05, ∗∗*p* < 0.01, ∗∗∗*p* < 0.001, and ∗∗∗∗*p* < 0.0001. (C) Flow cytometry analysis of T cell exhaustion markers (TIM-3, LAG-3, TIGIT) in tumor tissues across distinct treatment groups. Data are expressed as means ± SD (*n* = 3 mice).

To further explore the capacity and underlying mechanisms of _RGD_AB@DFX NPs in regulating tumor immunity, we designed a combinatorial therapeutic regimen incorporating anti-mouse PD-1 antibody (α-PD-1) RMP1-14. B16F1 tumor-bearing mice with tumor volumes of ∼100 mm^3^ were randomly allocated into four treatment cohorts: PBS control, α-PD-1 monotherapy, _RGD_AB@DFX NPs monotherapy, and α-PD-1 in combination with _RGD_AB@DFX NPs. Mice in each group received the corresponding agent via tail vein injection every other day for a total of three administrations. Following the completion of the treatment regimen, the proportion of CD8^+^ T cells infiltrating tumor tissues was quantified across all groups. As shown in Figure 7A, both the α-PD-1 and _RGD_AB@DFX monotherapy groups exhibited a significant increase in tumor-infiltrating CD8^+^ T cell percentages relative to the PBS group. Notably, the combinatorial treatment group achieved the most robust elevation, with CD8^+^ T cells accounting for 22.3 ± 1.5%, which was substantially higher than those of the PBS (8.4 ± 0.6%), α-PD-1 monotherapy (15.4 ± 0.7%), and _RGD_AB@DFX monotherapy (17.2 ± 0.8%) groups (Figure S4). Additionally, we measured the concentrations of downstream cytokines associated with immune activation in tumor tissues, including pro-inflammatory cytokines (interferon-γ (IFN-γ); tumor necrosis factor-α (TNF-α)) and the anti-inflammatory cytokine interleukin-10 (IL-10). As illustrated in Figure 7B, monotherapy with α-PD-1 or _RGD_AB@DFX, as well as their combination, enhanced IFN-γ and TNF-α secretion while suppressing IL-10 expression. Among these interventions, the combinatorial therapy induced the most profound alterations in cytokine profiles. This synergistic effect is attributed to a dual-blockade mechanism: _RGD_AB@DFX NPs downregulate PD-L1 expression on tumor cells, thereby reducing the availability of PD-L1 ligands for PD-1 binding; concurrently, α-PD-1 competitively blocks residual PD-1 binding sites on T cells. This dual inhibitory action effectively abrogates the immunosuppressive signaling that constrains T cell effector function.

To gain deeper mechanistic insights into T cell functionality, we performed flow cytometry to comprehensively analyze the expression of key T cell exhaustion markers (TIM-3, LAG-3, and TIGIT) on tumor-infiltrating T cells. The results demonstrated that both α-PD-1 and _RGD_AB@DFX monotherapies reduced the proportion of T cells positive for these exhaustion markers compared to the PBS group. Critically, the combination of α-PD-1 and _RGD_AB@DFX exerted the most potent effect in reversing T cell exhaustion (Figure 7C). Collectively, these findings definitively validate that our constructed nanotherapeutic platform effectively mitigates T cell exhaustion within the TME through the targeted modulation of the PD-1/PD-L1 signaling pathway.

## Discussion

In the clinical setting, therapeutic options for advanced melanoma remain remarkably limited, with ICB therapy representing one of the mainstay treatments for affected patients.^23^ PD-1 and PD-L1 are key immune checkpoint molecules that are responsible for the negative regulation of the stability and the integrity of T cell immune function.^24^ However, tumor cells exploit this pathway by upregulating PD-L1 expression, thereby suppressing T cell-mediated immune clearance.^25^ It is widely accepted that downregulating PD-L1 expression within the TME serves as a robust strategy to reprogram the immunosuppressive TME and restore T cell-mediated anti-tumor cytotoxicity.^26^^,^^27^^,^^28^ Accordingly, the rationale of this study focuses on Fe chelation and PI3K/AKT pathway suppression, which downregulate PD-L1 expression and alleviates the PD-L1/PD-1 interaction between tumor cells and T cells, ultimately reactivating T cells and restoring their cytotoxic function against tumor cells. As a highly aggressive cutaneous malignant tumor, melanoma exhibits prominent immunosuppressive features within its TME.^29^ Studies have demonstrated that melanoma cells display significantly higher intracellular Fe levels compared to normal cells, a phenomenon referred to as Fe overload.^30^ This condition not only supports the rapid proliferation and metastasis of tumor cells but also potentially activates the PI3K/AKT signaling pathway indirectly, leading to increased PD-L1 expression.^31^^,^^32^^,^^33^^,^^34^ Therefore, the targeted reduction of intracellular Fe content in tumor cells may effectively downregulate PD-L1 expression, thereby providing a promising direction for the development of immunotherapeutic strategies against melanoma.

DFX is an efficient Fe chelator approved by the U.S. FDA for treating chronic Fe overload. However, the inherent physicochemical limitations of DFX, such as its poor water solubility (<0.5 mg/mL), low oral bioavailability (∼40%), and lack of tumor-specific distribution, significantly inhibit its clinical application for the reduction of ion level.^35^^,^^36^ To address these challenges, this study innovatively constructed a “three-in-one” albumin-based nanodelivery system, _RGD_AB@DFX NPs, which achieved key functional optimizations through rational design. The system begins with an albumin core, where human serum albumin is utilized for its natural drug-binding capacity to efficiently encapsulate DFX molecules via the nanoprecipitation method, forming stable A@DFX NPs. This albumin core not only significantly enhances the water solubility of DFX but also provides active sites for subsequent functional modifications due to its abundant amino acid residues.^37^ Next, a disulfide crosslinking layer is introduced, employing BAC as an intelligent crosslinker to construct AB@DFX NPs with redox-responsive properties. This design offers dual advantages: The disulfide bonds enhance the stability of NPs in the bloodstream, while the high concentrations of GSH (2–10 mM) in tumor cells can specifically cleave the disulfide bonds, enabling triggered drug release.^38^ Finally, a cRGD targeting modification layer is incorporated, where maleimide-thiol is used to conjugate cyclic arginine-rich peptide to the surface of NPs, endowing the final product _RGD_AB@DFX NPs with active targeting capabilities. The cRGD peptide specifically recognizes αvβ3 integrin, which is overexpressed on tumor vasculature and cells.^39^ This integrated approach ensures the NPs’ functionality, stability, and tumor-targeting efficiency.

_RGD_AB@DFX NPs exhibited nanoscale size, high stability, and efficient DFX release under high GSH level. Upon specific internalization by B16F1 cells, _RGD_AB@DFX NPs could significantly inhibit *p*-AKT and PD-L1 expression. Following intravenous injection into B16F1 tumor-bearing mice, _RGD_AB@DFX NPs displayed a prolonged circulation half-life compared with free DFX and preferentially accumulated in tumor tissues, resulting in pronounced tumor growth inhibition after 21 days of treatment. Reduced PD-L1 expression in tumor tissues further confirmed that the therapeutic efficacy of _RGD_AB@DFX NPs was ascribed to DFX release and subsequent modulation of PI3K/AKT/PD-L1 signaling pathway. These results demonstrate that the successful delivery of DFX into tumor cells can reduce PD-L1 expression, providing a promising approach for improving tumor therapy.

To date, therapeutic antibodies targeting the PD-1/PD-L1 axis—including nivolumab, pembrolizumab, and BMS-936559—have been translated into clinical practice for melanoma treatment. However, these PD-1/PD-L1 blockers are still plagued by multiple limitations, such as suboptimal response rates in specific cancer subtypes, the lack of reliable predictive biomarkers, the occurrence of immune-related adverse events (irAEs), and the development of both intrinsic and acquired resistance. _RGD_AB@DFX NPs exhibit TME-responsive release and precise tumor cell targeting. By modulating Fe metabolism via DFX to endogenously downregulate tumor cell PD-L1 expression, this nanoplatform effectively circumvents the limitations of conventional PD-1/PD-L1 blockers, providing a promising strategy to overcome ICI resistance in advanced melanoma. Moreover, _RGD_AB@DFX NPs showed robust biocompatibility, as evidenced by negligible changes in body weight, hematological and biochemical parameters, and the absence of noticeable pathological abnormalities. Collectively, these findings demonstrate that _RGD_AB@DFX NPs offer an effective and biocompatible nanoplatform for melanoma therapy, holding great potential for future clinical translation.

### Limitations of the study

This study has yielded promising results, yet mechanistically related limitations require attention in future research. AP-1 is a well-established downstream target of signaling cascades including the PI3K/AKT pathway,^40^ and this molecular link subtly paves the way for future investigations. It raises the intriguing possibility that our Fe-chelation strategy may exert regulatory effects on other key transcriptional regulators such as AP-1, rather than being limited to PD-L1 alone. αvβ3 integrin-mediated targeting proved effective in melanoma, but its applicability to other cancer types with distinct integrin expression profiles—linked to the nanoplatform’s targeting specificity—remains unexamined. _RGD_AB@DFX NPs emerge as a versatile, biocompatible nanoplatform for melanoma therapy with implications for modulating Fe-dependent immune evasion pathways in cancer. Future studies should evaluate their synergy with immune checkpoint inhibitors such as anti-PD-1/PD-L1 antibodies to clarify whether combined therapy enhances antitumor immunity and uncover the interactive mechanisms between Fe metabolism regulation and checkpoint blockade. These investigations not only hold important theoretical significance but also offer new therapeutic options for clinical practice.

## Resource availability

### Lead contact

Requests for further information and resources should be directed to and will be fulfilled by the lead contact, Haiyuan Zhang (hzhang@gzhmu.edu.cn).

### Materials availability

There are no additional data, software, databases, or applications/tools available beyond those disclosed in the current study. All data are included in the article and supplementary data section.

### Data and code availability

• This article does not report original datasets.
• This article does not report original code.
• Any additional information required to reanalyze the data reported in this article is available from the lead contact upon request.

## Acknowledgments

This work was primarily supported by 10.13039/501100012166National Key R&D Program of China (2021YFE0100300, 2021YFF0704805) and Science and Technology Development Program Project of Jilin Province (20220101047JC).

## Author contributions

X.S.: investigation, data curation, writing original draft, and methodology. W.Z.: data curation, writing review and editing, and investigation. L.W.: supervision, formal analysis, and data curation. P.S.: supervision, resources, and formal analysis. X.W.: visualization, validation, methodology, and investigation. X.J.: investigation and conceptualization. H.Z.: supervision, conceptualization, funding acquisition, and project administration.

## Declaration of interests

The authors declare that they have no known competing financial interests or personal relationships that could have appeared to influence the work reported in this paper.

## STAR★Methods

### Key resources table

**Table:** 

REAGENT or RESOURCE	SOURCE	IDENTIFIER
**Antibodies**		

Anti-AKT Antibody	Abcam	Cat# ab18785; RRID: AB_302604
Anti-AKT (phospho S473) Antibody	Abcam	Cat# ab38449; RRID: AB_732550
Anti-PD-L1 Antibody	Abcam	Cat# ab252436; RRID: AB_2890031
Anti-GAPDH Antibody	Abcam	Cat# ab181602; RRID: AB_2630358
Anti-Mouse PD-1 Antibody (RMP1-14)	Thermo Fisher	Cat# 14-9982-82; RRID: AB_469327
Anti-Mouse CD3 Monoclonal Antibody	Thermo Fisher	Cat# 17-0031-82; RRID: AB_468659
Anti-Mouse CD8a Monoclonal Antibody	Thermo Fisher	Cat# 11-0081-85; RRID: AB_464931
Anti-Mouse CD366 (TIM-3) Monoclonal Antibody	Thermo Fisher	Cat# 17-5871-80; RRID: AB_469105
Anti-Mouse CD223 (LAG-3) Monoclonal Antibody	Thermo Fisher	Cat# 12-2231-82; RRID: AB_2572405
Anti-Mouse TIGIT Monoclonal Antibody	Thermo Fisher	Cat# 17-9500-82; RRID: AB_2571782

**Chemicals, peptides, and recombinant proteins**		

Bovine Serum Albumin (BSA)	Aladdin	Cat# B265993
Deferasirox (DFX)	Shanghai yuanye Bio-Technology Co., Ltd	Cat# B34600
cyclo(Arg-Gly-Asp-D-Tyr-Lys) (cRGDyK)	Synpeptide Co., Ltd	Cat# 04010006008
N, N′-Bis (acryloyl) cystamine (BAC)	Macklin	Cat# N836631
Sulfosuccinimidyl-4-(N-maleimidomethyl) cyclohexane-1-carboxylate (Sulfo-SMCC)	Macklin	Cat# N814388
2-Iminothiolane	Macklin	Cat# I805674
Glutaraldehyde	Macklin	Cat# G810415
RPMI 1640 medium	Thermo	Cat# A26824DJ
Penicillin/Streptomycin	Thermo	Cat# 15140122
(6)-Fluorescein Isothiocyanate (FITC)	Beyotime	Cat# ST2930
Dimethyl sulfoxide (DMSO)	Beyotime	Cat# ST2335
Calcein acetoxymethyl ester (calcein AM)	Beyotime	Cat# C1367L
Propidium iodide (PI)	Beyotime	Cat# C1063
Sodium hydroxide (NaOH)	Aladdin	Cat# S431793
Ethanol	Aladdin	Cat# E433211
Methyl *tert*-butyl ether	Aladdin	Cat# G649582
Glutathione (GSH)	Beyotime	Cat# Y270851
3-(4,5-Dimethylthiazol-2-yl)-2,5-diphenyltetrazolium bromide (MTT)	Beyotime	Cat# C0009

**Critical commercial assays**		

HPLC	Agilent	1260 Infinity III

**Experimental models: Cell lines**		

Mouse Melanoma Cells (B16F1)	ATCC	CRL-6323

**Experimental models: Organisms/strains**		

Mouse: C57BL/6J	Guangdong Medical Laboratory Animal Center	RRID:IMSR_JAX:000664

**Software and algorithms**		

ImageJ	NIH	https://imagej.nih.gov/ij/
GraphPad Prism	GraphPad Software	https://www.graphpad.com/
FlowJo (Version 10)	FlowJo LLC	https://www.flowjo.com/
SPSS Statistics	IBM	https://www.ibm.com/products/spss-statistics

### Experimental model and study participant details

#### Cell culture

Murine melanoma B16F1 cells were purchased from the American Type Culture Collection (ATCC, Manassas, VA, USA). The cells were cultured in DMEM medium supplemented with 10% fetal bovine serum (FBS) and 1% penicillin/streptomycin (P/S) at 37 °C in a humidified atmosphere containing 5% CO_2_. The cell line was authenticated by ATCC using their standard authentication procedures and tested negative for mycoplasma contamination.

#### Mouse experiments

All mouse experiments were performed to ensure the compliance and reliability of *in vivo* assays by using qualified experimental mice under standardized housing conditions and with approved ethical protocols. All male and female C57BL/6 mice (6–8 weeks old) were obtained from the Guangdong Medical Laboratory Animal Center. All animal experiments were carried out at Guangzhou Medical University. Animals were housed in a specific pathogen free (SPF) environment with free access to food and water. All animal care and experimental procedures were strictly performed in accordance with the guidelines approved by the Animal Research Ethics Committee of Guangzhou Medical University (Ethics Approval No.: GY2024-621).

### Method details

#### Preparation of A NPs

Desolvation method was used to prepare A NPs according to previous method.^41^ The pH value of 1 mL of 15 mg mL^−1^ BSA aqueous solution was adjusted to 8–9 using 0.2 mol L^−1^ sodium hydroxide (NaOH) solution. 2 mL of ethanol was added to above BSA solution at a rate of 1 mL min^−1^ under constant stirring. Then, 0.1 mL of 10 mg mL^−1^ glutaraldehyde ethanolic solution was added to the mixture to induce cross-linking. After 24 h of stirring at 50 °C, the mixture was centrifuged at 10000 rpm for 15 min, and the sediment as A NPs was washed, collected and re-suspended in water.

#### Preparation of A@DFX NPs and AB@DFX NPs

A@DFX NPs were prepared following the same procedure as described for A NPs except using DFX ethanolic solution instead of 2 mL of ethanol. AB@DFX NPs were prepared following the similar procedure as described for A@DFX NPs except using BAC ethanolic solution instead of glutaraldehyde ethanolic solution.

#### Preparation of _RGD_AB@DFX NPs

cRGD-BSA is synthesized using the method of Wang et al.^42^ First, 50 mg of BSA was dispersed in 10 mL of distilled water and activated with 3.65 mg of sulfo-SMCC for 2 h. At the same time, 3 mg of cRGDyK and 1.2 mg of 2-iminothiolane were reacted at 4°C in 5 mL distilled water for thiolation. Finally, the sulfo-SMCC-activated BSA and thiolated-cRGD were mixed for stirring at 4°C for 24 h. The mixture was purified by ultrafiltration with 10 kDa molecule weight cut-off (MWCO) filters and lyophilized to obtain cRGD-BSA, which was identified by sodium dodecyl sulfate polyacrylamide gel electrophoresis (SDS-PAGE) (Figure S1). _RGD_AB@DFX NPs were prepared following the similar procedure as described for AB@DFX NPs except using cRGD-BSA instead of BSA.

#### Physicochemical characterization

The hydrodynamic particle size, PDI and zeta potential of NPs were measured by a Malvern ZetaSizer Nano ZS analyzer (Nano-ZS ZEN3600, Malvern, UK). Primary size and shape of NPs were characterized using transmission electron microscope (Jeol JEM-1230, Japan). The concentration of DFX was determined using an Agilent 1260 high-performance liquid chromatography (HPLC) system equipped with an Eclipse Plus C18 column (4.60 mm × 250 mm, 5 μm) (Agilent, USA).

#### GSH-responsive drug release

The GSH-responsive DFX release from various NPs was investigated using a dialysis membrane method. In brief, 2 mL of 10 mg mL^−1^ NP aqueous suspension was transferred into a dialysis bag (MWCO 7000 Da) and dialyzed against PBS (10 mM, pH 7.4) at 10 mmol L^−1^ or 20 μmol L^−1^ GSH. The released DFX was determined by HPLC at different time points (1, 2, 4, 8, 12, 24 and 48 h). The cumulative release percentage of DFX was calculated according to the following formula^43^:

$$Q_{n}\left(\%\right)=\frac{\sum_{n=1}^{n-1}C_{n}V_{2}+C_{n}V_{1}}{M}\times 100\%$$

(Qn is the cumulative release rate corresponding to each time point, Cn is the concentration corresponding to each time point, M is the total amount of DFX contained in the NPs placed in the dialysis bag, V_1_ is the total volume of the release medium, V_2_ is the volume of the release medium is determined for each removal.)

#### Cell viability assay

The viability of B16F1 cells exposed to various NPs was measured by MTT assay. In brief, B16F1 cells were inoculated at a density of (5×10^3^/well) in 96 well plates for 24 h incubation. Then, the culture medium was removed, and 0.1 mL of fresh medium containing various NPs (equivalent to DFX content) was added to each well. After 24 h of treatment, 0.1 mL of MTT working solution (5 mg mL^−1^) was added to each well for 4 h incubation. Then, the solution was removed and DMSO was added to each well to dissolve formazan, followed by measurement of the absorbance at 490 nm to calculate the cell viability.

#### Cellular uptake

Cellular uptake was investigated by fluorescence microscope and flow cytometry using FITC-labeled NPs that were prepared using the similar procedure as described in DFX-loaded NP preparation except using FITC instead of DFX. In brief, B16F1 cells were inoculated at a density of (8×10^4^/well) in 6 well plates for 24 h incubation. Then, the medium was replaced with fresh medium containing 20 μg mL^−1^ FITC-labeled NPs (equivalent to FITC content) for 6 h at 37 °C. The cells were washed with PBS three times and visualized by a Nikon Ti-S fluorescence microscope.

#### Melanoma mouse model

To examine the *in vivo* antitumor effect of various NPs, melanoma-bearing mice models were established. All male and female C57BL/6 mice (6–8 weeks old) were obtained from the Guangdong Medical Laboratory Animal Center. After one week of acclimation, each mouse was subcutaneously injected with 100 μL of PBS containing 1 × 10^6^ B16F1 cells on the back. All animal experiments were conducted at Guangzhou Medical University. All procedures involving laboratory animals were performed in accordance with the protocols approved by the Animal Research Ethics Committee of Guangzhou Medical University (Ethics Approval No.: GY2024-621).

#### Pharmacokinetics

Pharmacokinetic was conducted in C57BL/6 mice. Mice were randomly divided into four groups (*n* = 3) and intravenously injected with DFX, A@DFX NPs, AB@DFX NPs and _RGD_AB@DFX NPs at equal dose of 10 mg/kg (equivalent to DFX content). After injection, blood samples were collected from the eye socket at predetermined time points of 0.0, 0.17, 0.5, 1, 2, 4, 8, 12 and 24 h. Blood was centrifuged at 3000 g for 10 min to obtain the plasma. DFX was analyzed by HPLC. Briefly, 100.0 μL of plasma was mixed with 1.0 mL methyl *tert*-butyl ether to precipitate protein under vigorous vortex for 2 min. The samples were centrifuged at 3000 rpm for 5 min. The supernatant was blown dry by nitrogen and re-dissolved by adding 100.0 μL of mobile phase. The supernatant was taken for HPLC detection.

#### *In vivo* biodistribution

When the tumor volume got to 100 mm^3^, B16F1 tumor-bearing mice were intravenously injected with DiR-labeled _RGD_AB NPs (_RGD_AB@DiR NPs), DiR-labeled AB NPs (AB@DiR NPs), DiR-labeled A NPs (A@DiR NPs) or DiR (equivalent to 1 mg DiR per kg), in which DiR-labeled NPs were prepared using the similar procedure as described in DFX-loaded NP preparation except using DiR instead of DFX. At 24 h post-injection, the mice were sacrificed, and the major organs (heart, liver, spleen, kidney, and lung) were harvested for fluorescence imaging using an Aniview system (BLT, China).

#### *In vivo* therapeutic effect

When the tumor volumes reached approximately 100 mm^3^, mice were randomly divided into six groups (*n* = 3), and intravenously injected with PBS, A NPs, DFX, A@DFX NPs, AB@DFX NPs, _RGD_AB@DFX NPs at an equivalent dose of 10 mg DFX per kg body weight. The treatment regimen was defined as once every other day for a total of three doses, with administrations scheduled on Day 0, Day 2, and Day 4, corresponding to an overall treatment duration of 4 days. During 21 days of treatment, the tumor volume and the body weight were monitored every other day. The tumor volume was measured by a caliper and calculated using the formula: tumor volume (mm^3^) = (length) × (width)^2^ × 0.5. At the end of treatment, blood was collected from the eyes for blood routine and blood biochemistry tests, and the mice were sacrificed, and major organs (heart, liver, spleen, lung, kidney, and tumor) were harvested from mice, fixed in 10% neutral buffered formalin, and embedded in paraffin for H&E staining.

#### Immunological assays

After treatment, tumor tissues were mechanically dissociated and enzymatically digested to prepare single-cell suspensions. Flow cytometry was then used to quantify the proportion of tumor-infiltrating CD8^+^ T cells and the surface expression levels of T cell exhaustion markers (TIM-3, LAG-3, TIGIT) on these cells. For flow cytometry staining, tumor-derived single-cell suspensions were incubated with a panel of fluorochrome-conjugated monoclonal antibodies targeting CD3, CD8, TIM-3, LAG-3 and TIGIT. Gating was applied to the CD3^+^ T cell population for quantifying marker-positive cell percentages. Data were acquired on a flow cytometer and analyzed using FlowJo software (Version 10). Commercially available mouse cytokine-specific enzyme-linked immunosorbent assay (ELISA) kits were used to measure immune-related cytokine concentrations in tumor homogenates following the manufacturers’ protocols. The assayed cytokines included IFN-γ, TNF-α, and IL-10.

#### Statistical analysis

All statistical evaluations were completed via SPSS software (version 19.0). Unless otherwise indicated, *in vitro* experiments were performed in triplicate, with data summarized as mean ± standard deviation (SD). Pairwise group comparisons relied on a two-tailed Student’s *t* test, whereas multi-group comparisons were analyzed using one-way ANOVA coupled with Tukey’s post hoc test. Statistical significance was established at *p* < 0.05 (∗), *p* < 0.01 (∗∗), *p* < 0.001 (∗∗∗), *p* < 0.0001 (∗∗∗∗) and *p* > 0.05 (not significant, ns).
