# Supplementary material for: Targeted delivery and controlled release of deferasirox for melanoma therapy
Source: iScience. 2026 Mar 10;29(4):115303. doi: 10.1016/j.isci.2026.115303 (PMC13049520; doi:10.1016/j.isci.2026.115303)
Supplement: Document S1. Figures S1–S4 [file mmc1.pdf]

iScience, Volume *29*

Supplemental information

Targeted delivery and controlled release of deferasirox for melanoma therapy

Xiaochen Su, Weitao Zhao, Lulu Wang, Panpan Song, Xingbo Wang, Xuefei Jin, and Haiyuan Zhang

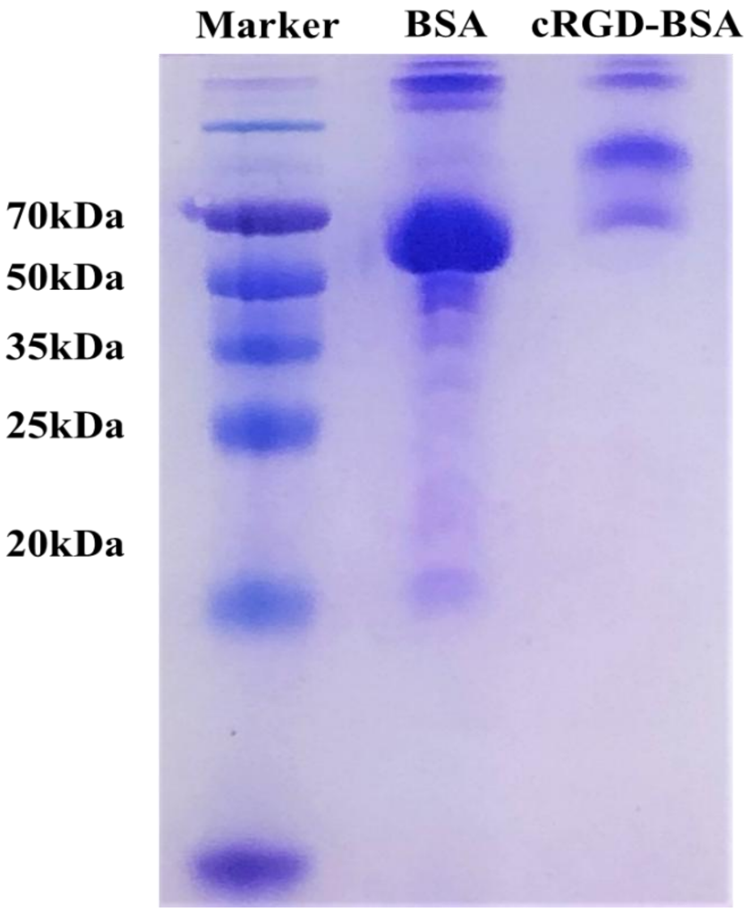

Figure S1. SDS-PAGE analysis of BSA and cRGD-BSA.

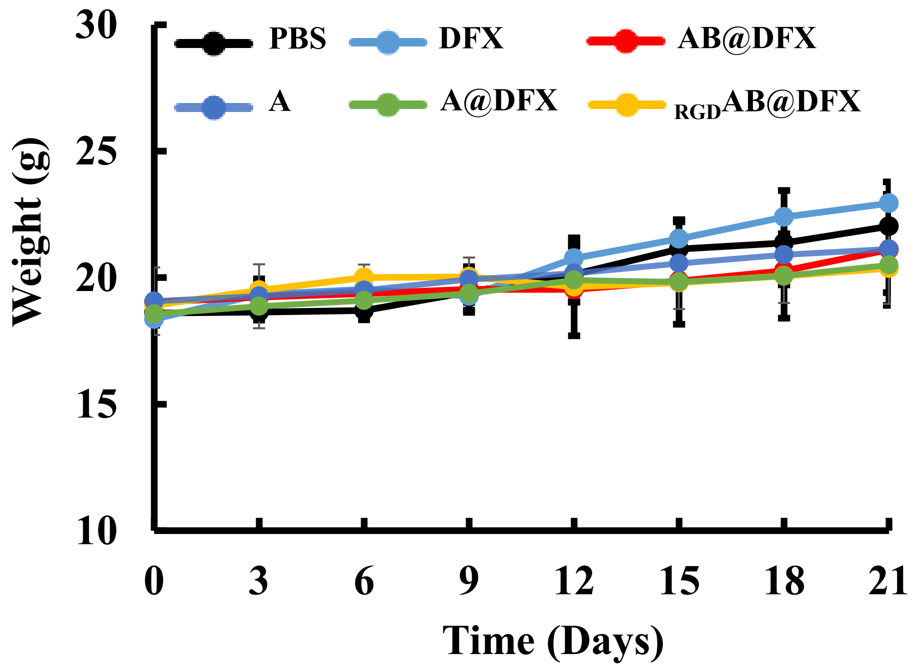

Figure S2. Body weights of mice during 21 days of treatments with PBS, DFX, A NPs, A@DFX NPs, AB@DFX NPs and $_{RGD}$AB@DFX NPs. Data are expressed as means ± SD (n=3 mice).

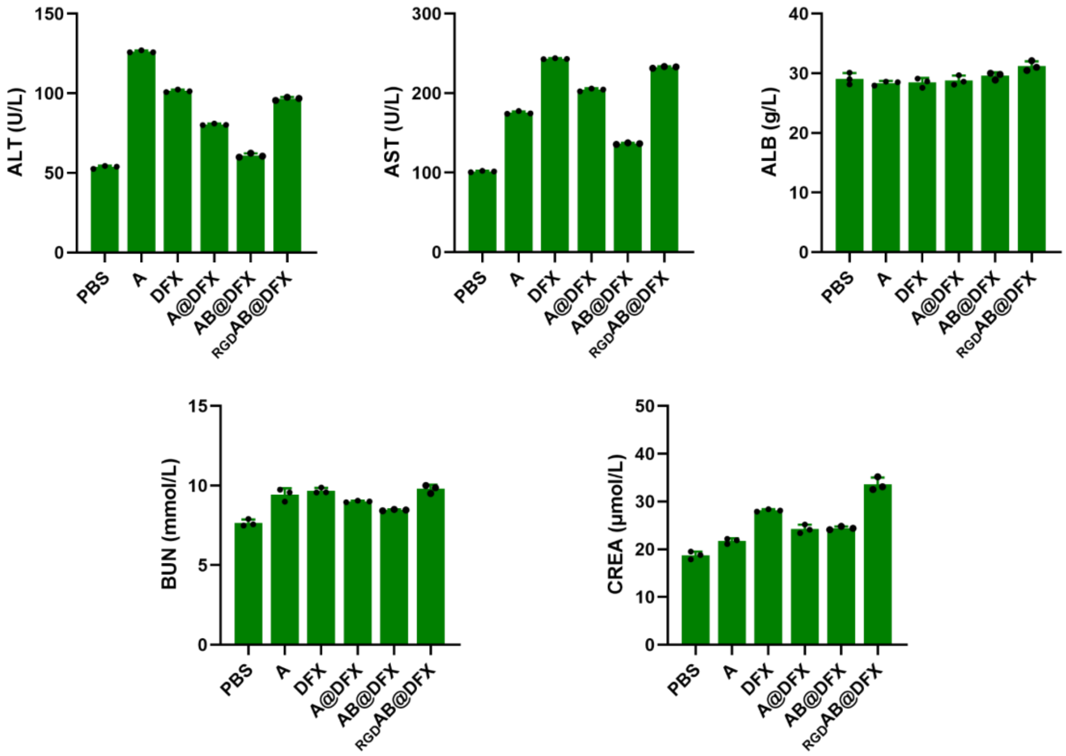

Figure S3. Blood biochemistry indices of mice at the end of 21 days of treatments with PBS, DFX, A NPs, A@DFX NPs, AB@DFX NPs and $_{RGD}$AB@DFX NPs. Data are expressed as means ± SD (n=3 mice).

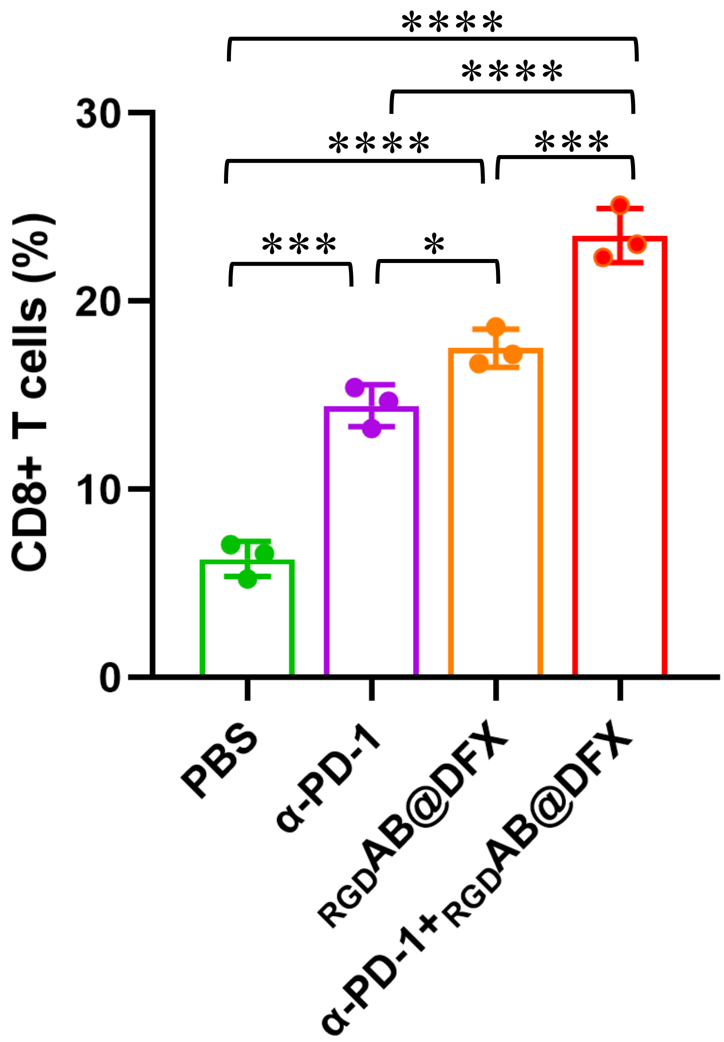

Figure S4. Proportion of $CD8^+$ T cells in tumor tissues among different groups. Data are expressed as means ± SD (n=3 mice). Statistical analysis: one-way ANOVA followed by Tukey's post hoc test. *$p < 0.05$, **$p < 0.01$, ***$p < 0.001$, ****$p < 0.0001$.
